# GENOME-WIDE ANALYSIS OF LOW STRENGTH IN OLDER PEOPLE: META-ANALYSIS OF >250,000 VOLUNTEERS IN 16 COHORTS

**DOI:** 10.1093/geroni/igz038.813

**Published:** 2019-11-08

**Authors:** Garan Jones, Luke C Pilling, David Melzer

**Affiliations:** 1 University of Exeter, Exeter, Devon, United Kingdom; 2 University of Exeter, Exeter, England, United Kingdom

## Abstract

Dynapenia or muscle weakness is a core feature of sarcopenia and frailty, and leads to significant functional impairment in older adults. We aimed to identify genetic variants associated with dynapenia in older adults, to shed light on the underlying mechanisms. A large-scale meta-analysis of >250,000 volunteers of European descent from 16 cohorts in the CHARGE and GEFOS consortia aged 60+ years at assessment is underway, using maximum hand grip strength to define dynapenia. Preliminary analysis in UK Biobank found that dynapenia is associated with nine genomic loci at genome wide significance (p<5x10-8), with differences between men and women, suggesting sex-specific routes to dynapenia. These results suggest that there are specific genetic determinants of low strength in older adults, and these differ from the determinants of normal-range strength differences. The full meta-analysis of all cohorts will extend these findings. Understanding the biological mechanisms may lead to geroscience interventions.

